# Temperature-sensitive auditory neuropathy: long-term follow-up and genotypic correlation

**DOI:** 10.1186/s13023-026-04500-5

**Published:** 2026-07-15

**Authors:** Xin Zhou, Qiujing Zhang, Xiaolong Zhang, Xiaonan Wu, Lan Lan, Qiuju Wang

**Affiliations:** 1 Department of Audio-Vestibular Medicine, Senior Department of Otolaryngology- Head and Neck Surgery, the Sixth Medical Center of Chinese PLA General Hospital, Beijing, 100048 China; 2 Department of Audio-Vestibular Medicine, Senior Department of Otolaryngology- Head and Neck Surgery, the First Medical Center of Chinese PLA General Hospital, Beijing, 100853 China; 3 State Key Laboratory of Hearing and Balance Science, Beijing, 100853 China; 4 National Clinical Research Center for Otolaryngologic Diseases, Medical School of Chinese PLA, Chinese PLA General Hospital, Beijing, 100853 China

**Keywords:** Temperature-sensitive auditory neuropathy, *OTOF*, Natural history

## Abstract

**Background:**

Temperature-sensitive auditory neuropathy (TSAN) is a rare disease and a distinctive subtype of auditory neuropathy, characterized by fluctuating hearing thresholds and speech recognition influenced by body or environmental temperature. It is most associated with biallelic variants in the *OTOF* gene. Despite growing genetic insights, its long-term natural history remains poorly characterized, and the functional consequences of specific *OTOF* variants are incompletely understood.

**Methods:**

This retrospective case series included 5 Han Chinese patients with TSAN carrying *OTOF* variants. Comprehensive audiological evaluations, including auditory brainstem response (ABR), otoacoustic emissions (OAE), cochlear microphonics (CM), pure-tone audiometry, and speech recognition assessment, were performed. Genetic analyses included whole-exome sequencing, Sanger validation, in silico pathogenicity prediction, and structural modeling. Long-term clinical follow-up ranged from 5 to 21 years.

**Results:**

All patients initially presented with fluctuating mild-to-profound hearing loss, absent ABR, and preserved OAE and/or CM, consistent with AN. Genetic analysis identified 7 rare *OTOF* variants, including 2 frameshift variants (p. Gln994Valfs*7 and p. Leu795Serfs*5), 1 nonsense variant (p. Arg1134Ter), and 4 missense variants (p. Arg1607Trp, p. Gly541Ser, p. Trp536Cys, and p. Ala1667Asp). Most variants affected evolutionarily conserved residues and were predicted to be deleterious, while structural modeling suggested that the missense variants may alter local protein conformation and stability. Long-term follow-up revealed marked inter-individual variability in auditory outcomes. Several patients achieved near-normal auditory performance without intervention, with Categories of Auditory Performance scores of 7 and Speech Intelligibility Rating scores of 5, whereas others showed persistent speech perception deficits despite only moderate hearing thresholds. Cochlear implantation improved auditory performance in one patient.

**Conclusions:**

*OTOF*-related TSAN demonstrated substantial clinical heterogeneity but generally favorable long-term functional outcomes in this cohort. These findings contribute to the characterization of the *OTOF* mutational landscape in TSAN, highlight the importance of long-term monitoring in this rare disease, and support individualized management strategies guided by both genotype and functional auditory performance.

**Supplementary Information:**

The online version contains supplementary material available at https://doi.org/10.1186/s13023-026-04500-5.

## Introduction

Auditory neuropathy (AN) is characterized by preserved outer hair cell function, evidenced by normal otoacoustic emissions (OAE) and/or cochlear microphonic (CM), together with absent or severely abnormal auditory brainstem responses (ABR), resulting in speech perception deficits disproportionate to pure-tone threshold loss [1, 2]. With further insights into auditory pathophysiology, AN is now recognized as a heterogeneous auditory processing disorder arising from dysfunction at multiple levels, including inner hair cells (IHCs), IHC ribbon synapses, spiral ganglion neurons, or demyelination and axonal loss of auditory nerve fibers and their central targets [3, 4].

Temperature-sensitive auditory neuropathy (TSAN) is a rare subtype of AN, in which hearing thresholds and speech recognition fluctuate with changes in body or environmental temperature [5]. This phenotype was first linked to biallelic variants in *OTOF*, which encodes otoferlin, a multi-C2 domain, calcium-sensing protein essential for synaptic vesicle exocytosis at IHC ribbon synapses [6, 7]. Otoferlin’s critical role in calcium-dependent neurotransmission means that even subtle structural changes can impair synaptic release, resulting in the characteristic electrophysiological findings of AN.

Recognizing TSAN is clinically important because its fluctuating symptoms can obscure the underlying disorder, delay diagnosis, or lead to misclassification as other hearing conditions [8]. However, longitudinal studies documenting its natural history are scarce, and whether TSAN can progress to irreversible AN remains uncertain. Despite growing knowledge of *OTOF*-related AN, significant gaps persist in understanding TSAN. Reported *OTOF* mutations encompass a broad range of variant types, including missense, nonsense, frameshift, and splice-site changes, but their functional consequences remain incompletely characterized. Although large-scale population databases show that most *OTOF* alleles are exceedingly rare (allele frequencies < 0.001), rarity alone does not explain the temperature-sensitive phenotype observed in some patients [9]. Furthermore, most previous studies have focused on individual case reports rather than investigating shared molecular mechanisms linking specific *OTOF* defects to temperature-dependent synaptic dysfunction.

To address these critical knowledge gaps, we investigated the long-term natural history and genetic mechanisms of TSAN in a cohort of five deeply phenotyped Han Chinese patients with TSAN carrying seven rare *OTOF* variants. These included two frameshift variants, one nonsense variant predicted to produce truncated proteins, and four missense variants located at highly conserved residues. Through detailed audiological evaluation, long-term follow-up, and comprehensive bioinformatic assessment, we contribute to the characterization of the *OTOF* mutational landscape in TSAN, refine genotype–phenotype correlations, and provide new evidence for counseling and management of affected individuals.

## Methods

### Participants and clinical evaluations

This study was approved by the Committee of Medical Ethics of Chinese People’s Liberation Army General Hospital (No. S2020-228-01) and conducted according to the Declaration of Helsinki. We obtained written informed consents from all the participants in this study. Five patients with TSAN were recruited, all with *OTOF* gene mutations (three males and two females). All participants underwent ABR and distortion product otoacoustic emissions (DPOAE) as the diagnostic examinations. Additional routine examinations included pure tone audiometry (PTA), acoustic immittance, speech discrimination scores (SDS), electrocochleography (ECochG), CM, and inner auditory canal magnetic resonance imaging (MRI). We conducted long-term follow-ups with the patients through face-to-face consultations at the hospital and telephone interviews. The follow-ups utilized standardized questionnaires, assessing auditory and speech abilities using categories of auditory performance (CAP) and speech intelligibility rating (SIR) classifications, and documented any new symptoms that appeared during the disease.

### Genetic sequencing and variant assessment

We collected the peripheral blood samples from five patients and their parents using EDTA anticoagulant tubes and extracted the genomic DNA using the Blood DNA kit (TIANGEN BIOTECH, Beijing, China). Trio-based whole exome sequencing (WES) and proband-only WES were then conducted for genetic sequencing, as described in detail previously [10]. Subsequently, Sanger sequencing was utilized to validate the candidate variants obtained from high-throughput sequencing.

### Bioinformatics analysis and variant pathogenicity assessment

High-frequency variants with an allele frequency greater than or equal to 0.01 in the normal population were eliminated, according to several large-scale population-based databases, including gnomAD. Bioinformatics analyses were carried out using the following tools, including Rare Exome Variant Ensemble Learner (REVEL), Sorting Intolerant From Tolerant (SIFT), Polymorphism Phenotyping (PolyPhen), MutationTaster, Phylogenetic P-Value (Phylop), Genomic Evolutionary Rate Profiling_Rejected Substitution (GERP++_RS) to predict the function of potential pathological variants.

We subsequently examined the conservation of the amino acid variants discovered in this study across different species using Uniprot and MutationTaster. We then annotated *OTOF* variants reported in our study, and labeled the spatial position of amino acid variants on the protein structure using PyMOL. The 3D structural model of the human *OTOF* protein was retrieved from the AlphaFold Protein Structure Database (ID: AF-Q9HC10-F1, model version 6). Finally, each variant was classified based on the American College of Medical Genetics and Genomics and the Association for Molecular Pathology (ACMG/AMP) criteria [11, 12] as pathogenic (P), likely pathogenic (LP), variants with uncertain significance (VUS), likely benign (LB), and benign (B).

## Results

### General characteristics

**Fig. 1:**
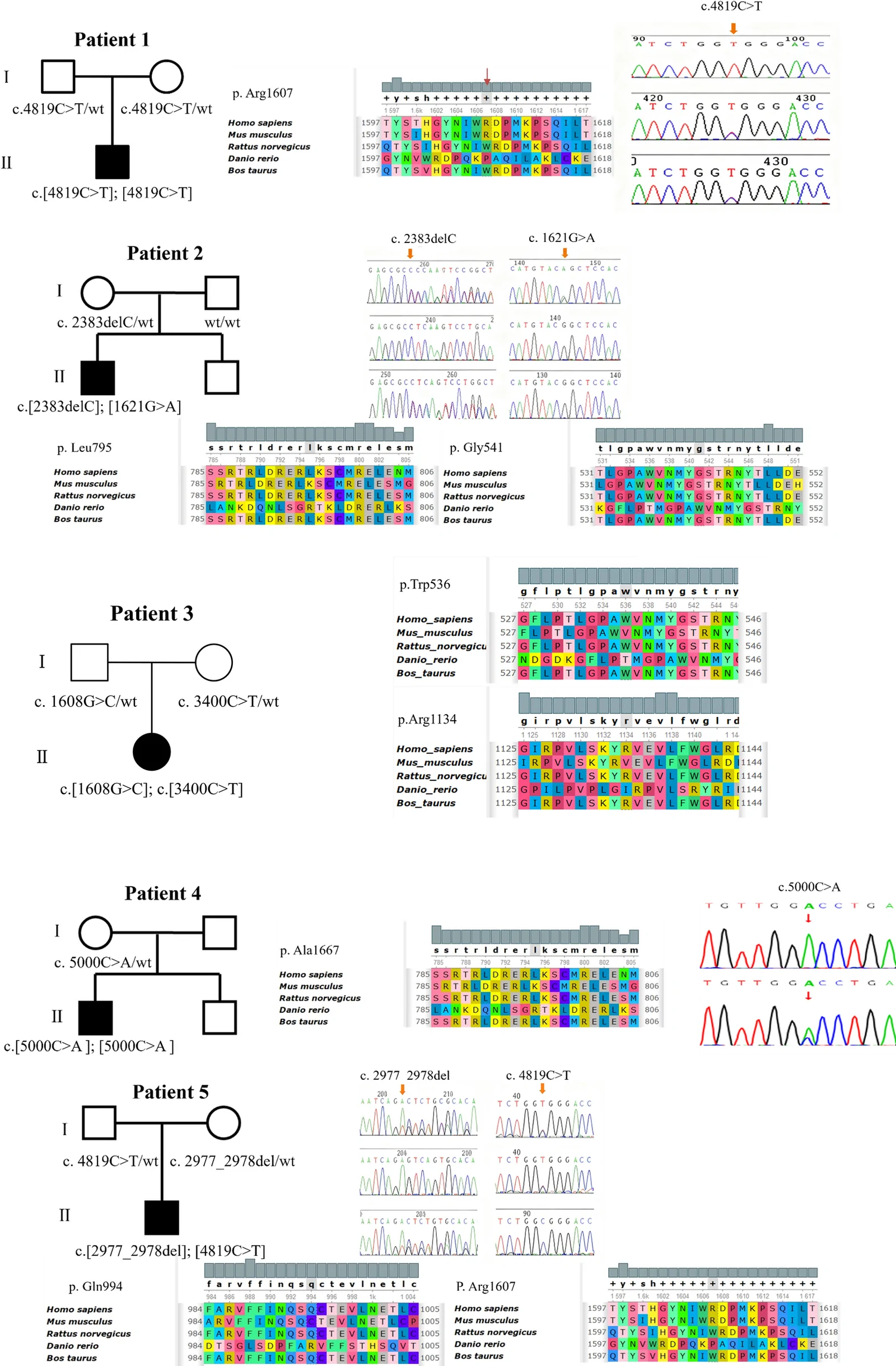
Pedigree, Sanger sequencing and interspecies sequence comparison of the five families included in this study. *OTOF* variants are reported according to NM_001287489.2, NP_001274418.1. Solid symbols indicate the affected probands, the grey background indicates the position of the mutated amino acid and arrows point to mutant bases. Sanger sequencing data for patient 3 were not available from the external laboratory and are therefore not shown

A total of 5 Han patients (3 males and 2 females) with TSAN from 5 families were included in this study (Fig. 1). Hearing loss was the initial symptom in all patients. The first diagnosis age of TSAN ranged from 3.5 to 6 years, with a mean age of diagnosis of 4.30 ± 0.87 years. Except for Patient 3, all patients had additional risk factors for hearing loss. Patient 1 had a history of gentamicin use. Patient 2 had breast milk jaundice appeared 2 days after birth and persisted for 40 days. At birth, patient 4 experienced hypoxia for about 4 h, had jaundice for 2–3 days, and her mother used gentamicin for 2 days during pregnancy. Patient 5 had severe jaundice, which persisted for 35 days. All patients, except for hearing issues, had no other systemic problems.

### Hearing evaluation and imaging examination

**Fig. 2:**
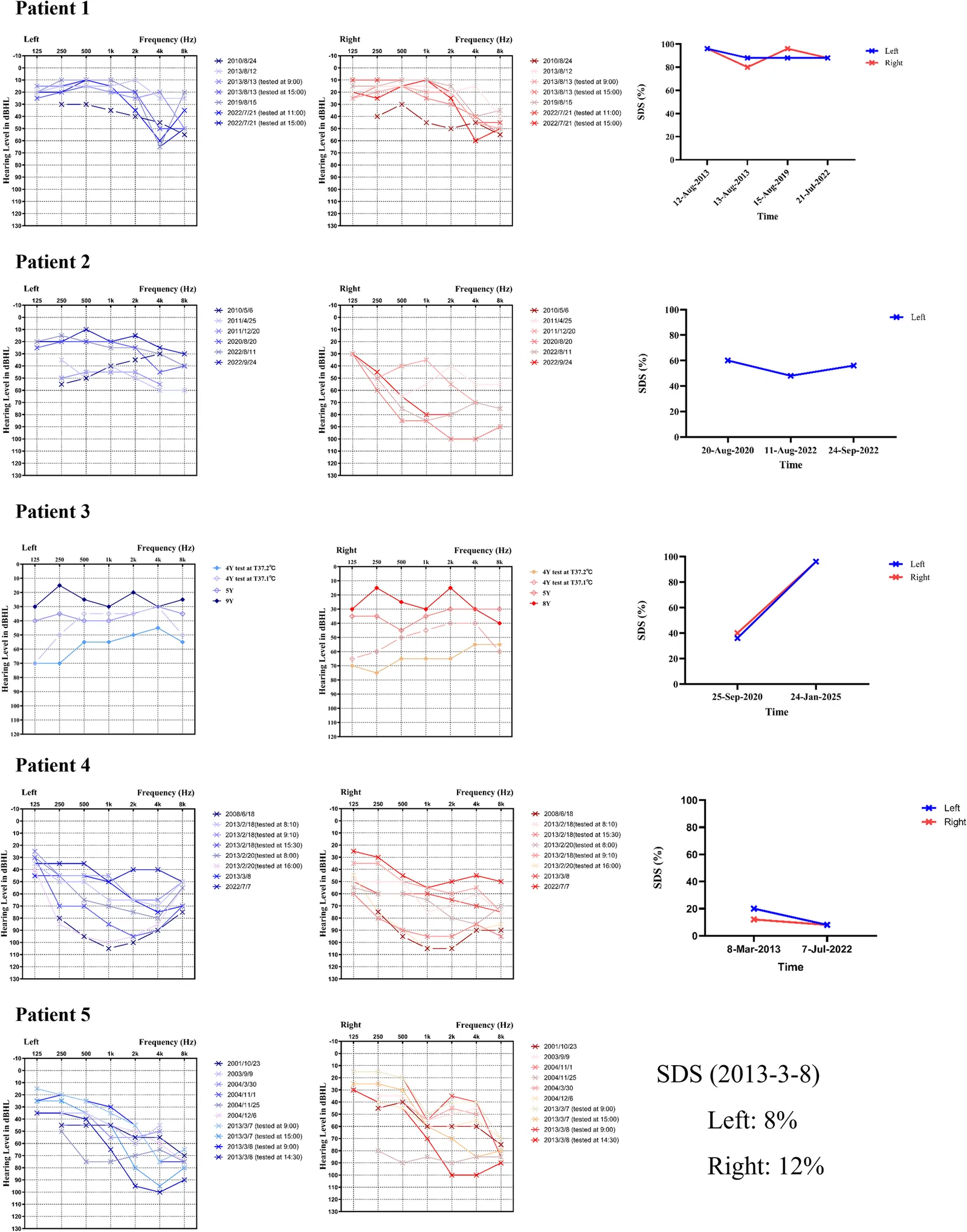
The characteristics of the subjective auditory tests of the patients. The left and middle columns show the progressive PTA thresholds for the left (blue lines) and right (red lines) ears, respectively, across various follow-up time points. The right column displays the corresponding longitudinal changes in SDS. PTA: pure-tone audiometry, SDS: speech discrimination score

All patients exhibited mild to profound hearing loss. The types of audiometric curves observed included flat, descending, and irregular patterns. Their speech recognition abilities were generally poorer than their hearing thresholds level (Fig. 2). All patients carried *OTOF* mutations and exhibited bilateral AN, with fluctuating pure tone audiometry indicating mild to profound hearing loss.

**Fig. 3:**
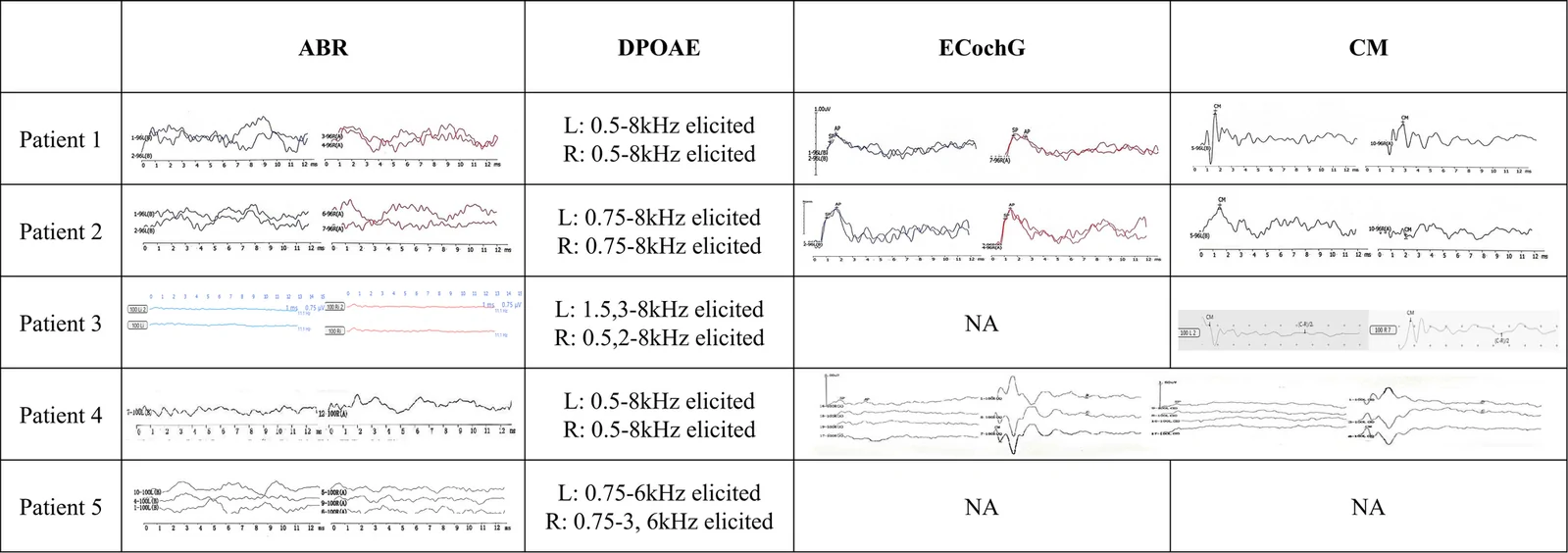
The characteristics of the objective auditory tests of the patients. DPOAE: distortion product otoacoustic emissions, ABR: auditory brainstem response, EcochG: Electrocochleography, CM: cochlear microphonic, SP: summating potential, AP: action potential. NA: not available

All patients underwent acoustic immittance tests and conductive problems were excluded. For the diagnostic audiological examination of AN, auditory brainstem response (ABR) waveforms could not be elicited, but distortion product otoacoustic emissions (DPOAE) could be elicited in the early stages of the disease. Among all patients, CM waves were elicited in 80% of cases. Additionally, 60% of patients underwent ECochG testing, exhibiting a reduced AP amplitude, resulting in an elevated summating potential SP/AP ratio with a mean of 0.94 ± 0.34. Across the entire patient group, the average SP amplitude was 0.24 ± 0.06µV, the average SP latency was 0.99 ± 0.10 ms, the average AP amplitude was 0.31 ± 0.07µV, and the AP latency averaged 1.86 ± 0.40 ms (Fig. 3).

**Fig. 4:**
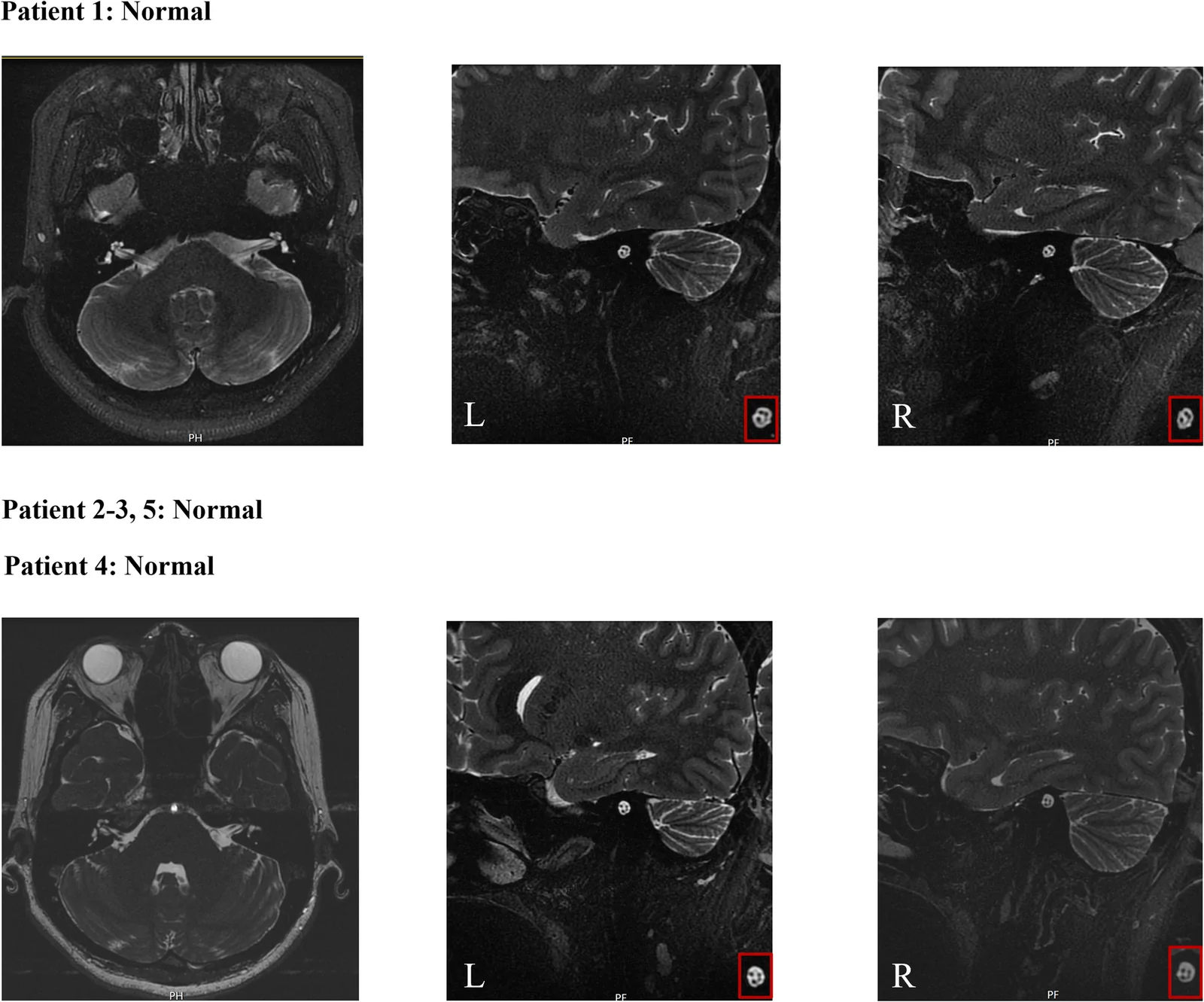
Representative MRI scans of the IAC and auditory nerves. The left column shows the axial high-resolution T2-weighted images. The middle and right columns show the oblique sagittal views of the left (L) and right (R) IAC. Red boxes in the lower right corners display the magnified cross-sectional views of the facial and vestibulocochlear nerves within the IAC, showing normal structures. IAC: internal auditory canal

All patients underwent MRI to observe the condition of their auditory nerves. No significant abnormalities were observed in all patients (Fig. 4).

### Follow-ups

All patients had an average follow-up period of 14.70 ± 5.38 years, with the shortest follow-up being 5 years and the longest being 21 years. Overall, hearing thresholds improved or stabilized in all patients, with improvements ranging from 6.25 to 56.25 dB HL. Patient 4 showed the most substantial recovery, from profound deafness bilaterally to moderate hearing loss, while Patients 1, 2, and 3 achieved mild or near-normal thresholds at the latest evaluation. Despite this general improvement in auditory sensitivity, speech recognition outcomes were highly variable. Three patients achieved near-normal daily auditory performance, with CAP scores of 7 and SIR scores of 4 to 5. Patient 1, for example, reported completely normal hearing in quiet environments with only slight difficulty in noise, and no longer experiences temperature-related fluctuations during activities such as hot showers. Patient 3 demonstrated marked spontaneous improvement from moderate to severe hearing loss to mild loss with essentially normal speech recognition. However, speech perception deficits persisted in some individuals despite improved pure-tone thresholds. Patient 4, whose PTA improved by over 50 dB HL bilaterally, attained only 8% speech recognition, and Patient 5, who showed some threshold improvement, maintained speech recognition rates of only 8 to 12% even during periods of better hearing. Temperature sensitivity showed variable persistence over time: Patient 2’s left ear continued to exhibit diurnal hearing fluctuation (better in the morning, worse in the evening) years after diagnosis, while Patient 1’s temperature-related fluctuations had fully resolved by adulthood. Patient 5 continued to show pronounced diurnal threshold shifts, with hearing ranging from mild-to-moderate in the morning to moderate-to-severe in the afternoon. Only Patient 2 underwent cochlear implantation in right ear, with documented benefit (CAP 7, SIR 5). All patients reported overall satisfaction with their communication abilities in daily life, and most were pursuing education or employment commensurate with their age, suggesting generally favorable long-term functional outcomes despite the persistence of objective audiological abnormalities. Detailed individual data are presented in Table 1.

**Table 1:** Overview of patients in this study on genotype and phenotype

Characteristic	OTOF (NM_001287489.2; NP_001274418.1)				
	Patient 1	Patient 2	Patient 3	Patient 4	Patient 5
Gender	M	M	F	F	M
Age at 2025	21	18	9	22	25
First diagnosis age of TSAN	6	3.5	4	4	4
ABR	NR	NR	NR	NR	NR
DPOAE	elicited	elicited	elicited	elicited	elicited
PTA (L/R) /dB HL	42.5/37.5	50/53.75	51.25/62.5	97.5/98.75	50/55
Nucleotide change	c. 4819 C > T	c. 2383delC	c. 1608G > C	c. 5000 C > A	c. 2977_2978del
	c. 4819 C > T	c. 1621G > A	c. 3400 C > T	c. 5000 C > A	c. 4819 C > T
Amino acid change	p. Arg1607Trp	p. Leu795Serfs*5	p. Trp536Cys	p. Ala1667Asp	p. Gln994Valfs*7
	p. Arg1607Trp	p. Gly541Ser	P. Arg1134Ter	p. Ala1667Asp	P. Arg1607Trp
ACMG classification	LP	P	LP	LP	P
		LP			LP
			P		
Pathogenic evidence	PM3_Strong＋PP3＋PP4	PVS1 + PM2+PP4	PM2+PM3 + PP3+PP4	PM2+PM3+PP1+PP4	PVS1 + PM2+PM3_Supporting+PP4
		PM2+PM3_Strong+PP4			PM3_Strong＋PP3＋PP4
			PVS1+ PM2 + PP4		
Follow-up years	15	14.5	5	18	21
Follow-up and the last ABR	Stable(NR)	Stable (NR)	Stable (NR)	Stable (NR with CM)	Stable (NR)
Follow-up and the last DPOAE	Stable (elicited)	Stable (L: elicited, R: CI)	Stable (elicited)	Stable (elicited)	Stable (elicited)
The last PTA (L/R)/dB HL	21.25/30	17.5/75.75(CI)	26.25/25	41.25/48.75	43.75/37.5
Hearing threshold improvement/dB HL	21.25/7.5	32.5/CI	25/37.5	56.25/50	6.25/17.5
Associated symptoms	None	None	None	None	None
Current work or study status	Sophomore in college	Second-year high school student	A student in a regular primary school	Post-graduation internship	Post-graduation internship
The patient is or is not satisfied with their communication and daily life	Satisfied	Satisfied	Satisfied	Satisfied with AR smart glass	Satisfied
CAP	7	7	7	None	6
SIR	5	5	4	None	4

## High-throughput sequencing and Sanger sequencing

**Fig. 5:**
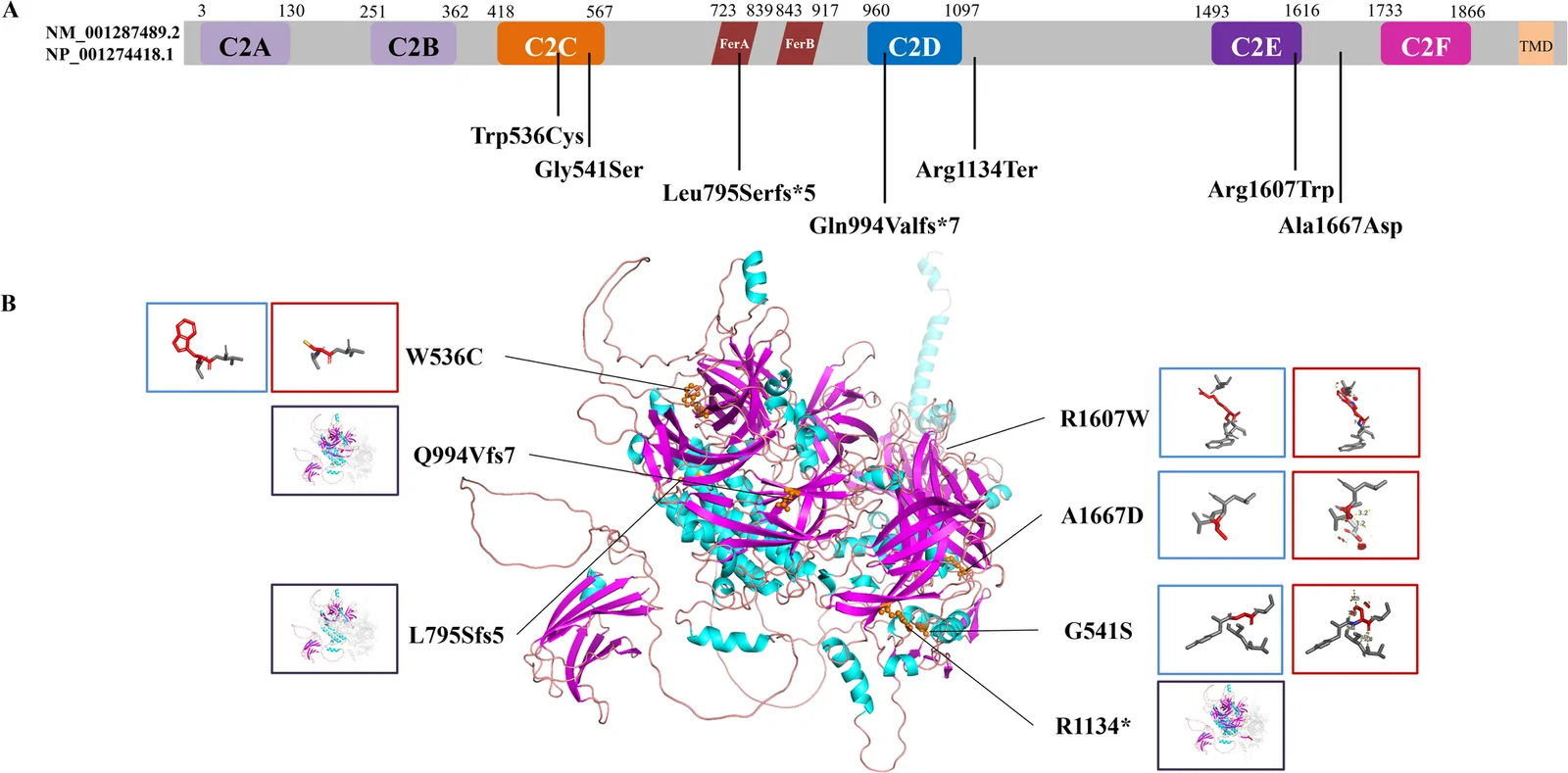
Distribution of *OTOF* variants identified in this study and their predicted structural consequences. (**A**) Structure of the *OTOF* gene and locations of variants (**B**) Three-dimensional model of otoferlin showing the spatial distribution of the identified variants. Secondary-structure elements are colored cyan for α-helices, magenta for β-strands, and light pink for loops/coils. For missense variants, boxed insets show the local conformations of the wild-type and mutant residues (blue and red frames, respectively). For truncating variants, including the frameshift variants L795Sfs*5 and Q994Vfs*7 and the nonsense variant R1134*, the downstream regions are depicted as structurally lost, reflecting the predicted disruption of the native C-terminal structure

Through high-throughput sequencing and Sanger sequencing, we identified a total of seven *OTOF* variants in five patients. Among these seven variants, four were missense variants (p. Arg1607Trp, p. Gly541Ser, p. Trp536Cys, and p. Ala1667Asp) and one nonsense variant (p. Arg1134Ter), with p. Gly541Ser located in the C_2_C domain; two were frameshift variants, in which p. Leu795Serfs*5 located in the FerA domain and p. Gln994Valfs*7 located within the C_2_D domain (Fig. 5). Overview of patients in this study on genotype and phenotype is presented in Table 1.

### Variant detection and analysis

Among all variants, four missense variants (p. Arg1607Trp, p. Gly541Ser, p. Trp536Cys, and p. Ala1667Asp) occur at evolutionarily conserved residues and were predicted to be deleterious by multiple in silico tools. p. Gln994Valfs*7 and p. Leu795Serfs*5 are frameshift variants and one nonsense mutation (p. Arg1134Ter) predicted to introduce premature termination codons and potentially trigger nonsense-mediated mRNA decay (NMD). All variants were re-classified according to ACMG and Bayes’ rule [13]. All other variants were classified as LP or P. The allele frequencies of all variants in gnomAD were < 0.001 in Supplementary 1.

## Discussion

In this study, we characterized the long-term natural history and genetic features of five deeply phenotyped Han Chinese patients with *OTOF*-related TSAN, a rare auditory orphan disease. Our principal contributions are threefold. First, we documented longitudinal clinical trajectories over up to 21 years, providing essential natural history data that have been absent from the TSAN literature. Second, we refined genotype–phenotype correlations by mapping the identified *OTOF* variants to specific protein structural domains and predicting their functional consequences. Third, we provided evidence to support individualized management strategies for this rare condition.

Clinically, all patients presented with early-onset auditory neuropathy characterized by mild to profound hearing loss, fluctuating thresholds, and disproportionately reduced speech recognition, consistent with previous descriptions of TSAN. Notably, longitudinal follow-up over up to two decades revealed that hearing thresholds in several patients improved or remained stable, whereas speech perception deficits persisted in some individuals. This dissociation between auditory sensitivity and speech recognition highlights the synaptic origin of dysfunction in *OTOF*-related AN. Given that the mutation frequency of *OTOF* in Chinese infants and young children with AN has been reported to be as high as 41.2% [14], recognizing its distinctive subtypes is of great clinical significance in this population. Fluctuations in hearing thresholds and speech discrimination, especially under varying body or ambient temperatures, were defining features of TSAN. For instance, Patient 2 demonstrated time-of-day dependent variations in hearing performance years after diagnosis, suggesting that synaptic dysfunction in TSAN remains temperature-sensitive long after onset. These findings are consistent with studies reporting that TSAN represents a unique clinical course caused by *OTOF* gene variants [15].

A key finding of this study is the relatively favorable long-term auditory outcome observed in most patients. Despite early auditory dysfunction, several individuals achieved near-normal auditory performance in daily life without cochlear implantation, as reflected by high CAP and SIR scores. These findings suggest that partial preservation of otoferlin function may reflect compensatory mechanisms or neural plasticity over time. However, inter-individual variability was evident. For example, one patient exhibited persistently poor speech recognition despite only moderate hearing thresholds, underscoring the clinical heterogeneity of TSAN. Together, these observations indicate that TSAN does not follow a uniform natural history and highlight the importance of individualized longitudinal monitoring.

Genetically, we identified four missense variants (p. Arg1607Trp, p. Gly541Ser, p. Trp536Cys, and p. Ala1667Asp), one nonsense variant (p. Arg1134Ter), and two frameshift variants (p. Leu795Serfs*5 and p. Gln994Valfs*7). The truncating variants are predicted to introduce premature termination codons and may trigger nonsense-mediated mRNA decay, which is consistent with the established loss-of-function mechanism of *OTOF*-related AN [16, 17]. Frameshift alleles in *OTOF* are widely recognized as LP and P under ACMG/AMP guidelines, given that loss of function is a well-established disease mechanism [8, 15, 18, 19].

The missense variants were located at evolutionarily conserved residues and were predicted to be deleterious by multiple in silico tools, suggesting potential functional impact on otoferlin. Our structural modeling suggests that these substitutions likely alter local folding, hydrogen bonding, or surface charge within these Ca²⁺-sensing domains. Given the critical role of otoferlin’s C2 domains in Ca²⁺-dependent synaptic vesicle tethering and fusion [20], even subtle conformational changes could impair synaptic transmission efficiency in a temperature-dependent manner, providing a plausible structural basis for the fluctuating phenotype. In addition, TSAN caused by *OTOF* mutations has been reported, where elevated temperature exacerbates synaptic transmission failure [21, 22]. Their extremely low allele frequencies (< 0.001 in gnomAD and 1KGP) further supports clinical relevance.

The molecular mechanisms underlying the temperature dependence of TSAN remain incompletely understood. Clinical and experimental observations indicate that elevated temperature can aggravate synaptic transmission failure in *OTOF*-related AN [15, 23]. This effect is presumed to arise from temperature-induced reductions in otoferlin stability or Ca²⁺ triggered vesicle release efficiency, although direct biophysical evidence is lacking. A study highlighted that a human-specific arginine-rich 20-amino acid stretch containing an RXR motif can, in combination with the p.Ile515Thr mutation, reduce membrane localization of otoferlin in inner hair cells, thereby impairing synaptic sound encoding at elevated temperature [23]. These findings provide a potential molecular basis for the heat-exacerbated phenotype observed in TSAN. Missense variants may therefore partially impair otoferlin’s synaptic function, leading to reversible dysfunction under thermal stress but near-normal activity at physiological temperatures.

Clinically, our findings highlight the importance of genetic testing in patients with AN, particularly those with fluctuating hearing and disproportionate speech recognition deficits. Early identification of *OTOF* variants can facilitate accurate diagnosis, avoid misclassification, and inform individualized management strategies. While cochlear implantation remains effective for patients with severe dysfunction, conservative management may be appropriate in patients with stable auditory performance, emphasizing the need for personalized follow-up.

### Limitations and future directions

First, the sample size of our cohort is relatively small. While this is inherent to the extreme rarity of *OTOF*-related TSAN, it restricts the statistical power to draw generalized genotype-phenotype conclusions across the broader patient population. Second, although we utilized comprehensive in silico predictions and 3D structural modeling to assess the impact of the identified *OTOF* variants, our study lacks direct functional validation. Future in vitro biophysical studies, such as patch-clamp recordings in hair cell models, or in vivo animal studies are required to definitively validate how these specific structural alterations in otoferlin directly cause temperature-dependent synaptic transmission failure. Despite these limitations, our study provides unprecedented long-term observational data that significantly enrich the clinical understanding of this rare auditory disorder.

## Conclusion

Our findings reinforce the strong association between *OTOF* mutations and TSAN, broadening the known *OTOF* mutational spectrum in the Chinese population. The diverse long-term outcomes observed, from near-normal hearing to persistent speech perception deficits, underscore the clinical heterogeneity of this condition. Our integration of longitudinal clinical data with protein structural modeling provides a tentative framework for understanding how specific missense variants may confer temperature-dependent synaptic dysfunction. Future studies combining in vitro biophysical characterization of these mutants with advanced electrophysiological assays will be essential for validating these mechanisms. By contributing essential natural history data and variant-level insights, this study provides valuable evidence to aid diagnostic accuracy, prognostic counseling, and the development of personalized management strategies for individuals with TSAN.

## Supplementary Information

Below is the link to the electronic supplementary material.

Supplementary Material 1

## Data Availability

No datasets were generated or analysed during the current study.
