# Supplementary material for: Temperature-sensitive auditory neuropathy: long-term follow-up and genotypic correlation
Source: Orphanet J Rare Dis. 2026 Jul 15;21:387. doi: 10.1186/s13023-026-04500-5 (PMC13732856; doi:10.1186/s13023-026-04500-5)
Supplement: Supplementary file 1 [file 13023_2026_4500_MOESM1_ESM.docx]

**Supplementary Table 1. The detailed information of gene testing for 5 subjects with Temperature-Sensitive AN**

| **Subjects** | **Diagnosis** | **Gene** | **Inheritance pattern** | **Homozygous/heterozygous** | **Genomic position** **(GRCh37/hg19)** | **Nucleotide change (Amino acid change)** | **ACMG classification** | **Evidence of** | **First diagnosis age of TSAN** | **Gender** | **DOB** | **Date of gene testing** |
| --- | --- | --- | --- | --- | --- | --- | --- | --- | --- | --- | --- | --- |
|  |  |  |  |  |  |  |  | **pathogenicity** |  |  |  |  |
| 1 | TSAN | *OTOF* | AR | Hom | chr2:26687878 | c. 4819C>T(p. Arg1607Trp) | LP | PM3_Strong＋PP3＋PP4 | 6 years old | male | 2004/5/22 | 2025/7/28 |
| 2 | TSAN | *OTOF* | AR | Het | chr2:26700307 | c. 2383delC (p. Leu795Serfs*5) | P | PVS1+PM2+PP4 | 3.5 years old | male | 2007/12/12 | 2022/9/7 |
| 2 | TSAN | *OTOF* | AR | Het | chr2:26703836 | c. 1621G>A (p. Gly541Ser) | LP | PM2+PM3_Strong+PP4 |  |  |  |  |
| 3 | TSAN | *OTOF* | AR | Het | chr2:26703849 | c. 1608G>C (p.Trp536Cys) | LP | PM2＋PM3＋PP3＋PP4 | 4 years old | female | 2016/7/14 | 2020/9/11 |
| 3 | TSAN | *OTOF* | AR | Het | chr2:26696867 | c. 3400C>T (p.Arg1134Ter) | P | PVS1+PM2+PP4 |  |  |  |  |
| 4 | TSAN | *OTOF* | AR | Hom | chr2:26686935 | c. 5000C>A (p. Ala1667Asp) | LP | PM2+PM3+PP1+PP4 | 4 years old | female | 2003/11/1 | 2022/8/11 |
| 5 | TSAN | *OTOF* | AR | Het | chr2:26698795-26698796 | c. 2977_2978del (p. Gln994ValfsTer7) | P | PVS1+PM2+PM3_Supporting+PP4 | 4 years old | male | 2000/10/9 | 2025/7/28 |
| 5 | TSAN | *OTOF* | AR | Het | chr2:26687878 | c. 4819C>T (p. Arg1607Trp) | LP | PM3_Strong＋PP3＋PP4 |  |  |  |  |

**Supplementary Table 2: *OTOF* mutations in this study**

| Nucleotide Change | Protein Change | Consequence | Impact | Bioinformatic Prediction Tools | | | | | |  | Frequency |
| --- | --- | --- | --- | --- | --- | --- | --- | --- | --- | --- | --- |
|  |  |  |  | REVEL | SIFT | Polyphen | MutationTaster | PhyloP100way vertebrate | GERP++_RS | CADD Phred | GnomAD |
| c. 4819C>T | p. Arg1607Trp | missense_variant | Moderate | 0.746 | Deleterious | Damaging | Disease Causing | Conserved (0.90) | Conserved (1.59) | 25.2 | 4.49E-06 |
| c. 2383delC | p. Leu795Serfs*5 | frameshift_variant | High | - | - | - | - | - | - | - | - |
| c. 1621G>A | p. Gly541Ser | missense_variant | Moderate | 0.872 | Deleterious | Probably Damaging | Disease Causing Automatic | Conserved (7.85) | Conserved (4.27) | 27.8 | 4.11E-06 |
| c. 1608G>C | p.Trp536Cys | missense_variant | Moderate | 0.906 | Deleterious | Probably Damaging | Disease Causing | Conserved (7.85) | Conserved (5.16) | 30 | - |
| c. 3400C>T | p.Arg1134Ter | stop_gained | High | - | - | - | Disease Causing Automatic | Conserved (1.37) | Conserved (2.51) | 40 | 1.83E-05 |
| c. 5000C>A | p. Ala1667Asp | missense_variant | Moderate | 0.263 | Deleterious | Possibly Damaging | Polymorphism | Conserved (5.17) | Conserved (4.4) | 25 | 0.00E+00 |
| c. 2977_2978del | p. Gln994Valfs*7 | frameshift_variant | High | - | - | - | - | - | - | 37 | - |

REVEL = Rare Exome Variant Ensemble Learner, SIFT = Sorting Intolerant from Tolerant, PolyPhen = Polymorphism Phenotyping, GERP++_RS = Genomic Evolutionary Rate Profiling_Rejected Substitution.
